# 773. Hypochlorous Acid Generating Electrochemical Catheter Prototype for Prevention of Intraluminal Infections

**DOI:** 10.1093/ofid/ofab466.970

**Published:** 2021-12-04

**Authors:** Laure Flurin, Edison J Cano Cevallos, Abdelrhman Mohamed, Kerryl Greenwood-Quaintance, Yash Raval, Haluk Beyenal, Robin Patel, Robin Patel

**Affiliations:** 1 Mayo Clinic, Rochester, Minnesota; 2 Washington State University, Pullman, Washington

## Abstract

**Background:**

Central-line associated bloodstream infection (CLABSI) contributes to mortality and cost. While aseptic dressings and antibiotic-impregnated catheters can prevent extraluminal infections, intraluminal infections remain a source of CLABSIs with limited prevention options.

**Methods:**

In this proof-of-concept study, an electrochemical intravascular catheter (e-catheter) prototype capable of electrochemically generating hypochlorous acid intraluminally on the surface of platinum electrodes polarized at a constant potential of 1.5 VAg/AgCl was developed. After 24h of pre-polarization at 1.5 VAg/AgCl, their activity was tested by inoculating four clinical isolates derived from catheter-related infections, *Staphylococcus aureus*, *Staphylococcus epidermidis*, *Enterococcus faecium* and *Escherichia coli*.

Figure 1. In vitro catheter and e-catheter models.

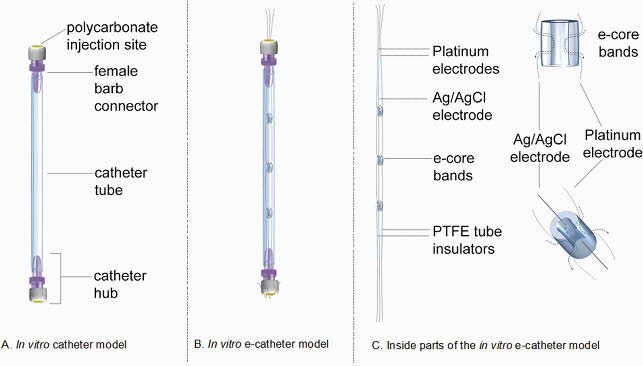

**Results:**

E-catheters generated a mean HOCl concentration of 15.86±4.03 μM and had a mean pH of 6.14±0.79. e-catheters prevented infections with all four species, with an average reduction of 8.41±0.61 log10 CFU/mL at 48h compared to controls.

Figure 3. Measurement of pH and HOCl at 48 hours in polarized e-catheters.

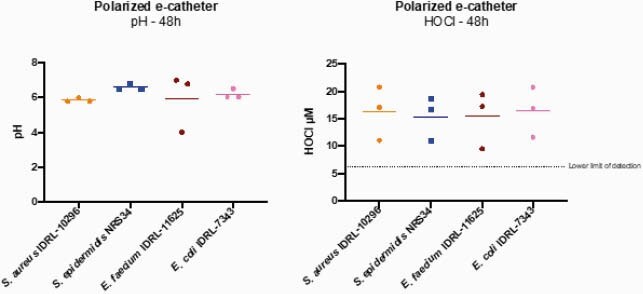

Each dot represents a replicate; bars represent means.

Figure 4. Prevention of infection after 48 hours of polarization (24 hours of infections) using e-catheters (polarized and non-polarized) compared to blank catheters.

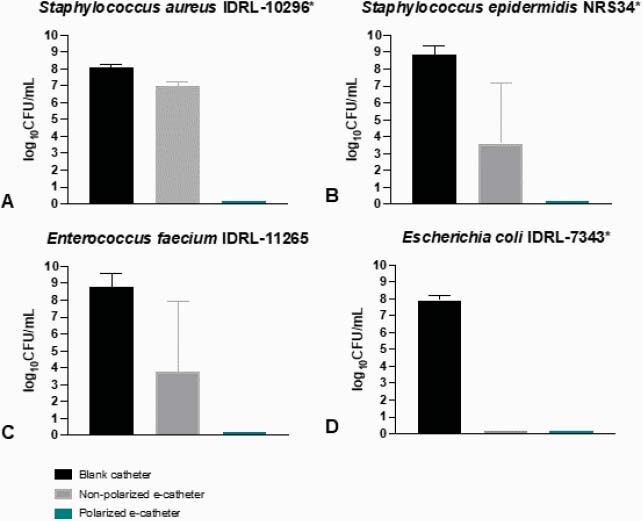

* indicates statistically significant reduction of cell counts in polarized e-catheter compared to blank catheter (p <0.05).

**Conclusion:**

Polarized e-catheters which generate low amounts of HOCl continuously should be further developed to prevent intraluminal infection.

**Disclosures:**

**Haluk Beyenal, Ph.D**, **patent** (Other Financial or Material Support, HB holds a patent: Beyenal H CD, Fransson BA, Sultana ST. . 2018. Electrochemical reduction or prevention of infections. U.S. patent 20180207301A1, international patent WO/2017/011635.) **Robin Patel, MD**, **1928 Diagnostics** (Consultant)**BioFire Diagnostics** (Grant/Research Support)**ContraFect Corporation** (Grant/Research Support)**Curetis** (Consultant)**Hylomorph AG** (Grant/Research Support)**IDSA** (Other Financial or Material Support, Editor's Stipend)**Infectious Diseases Board Review Course** (Other Financial or Material Support, Honoraria)**Mammoth Biosciences** (Consultant)**NBME** (Other Financial or Material Support, Honoraria)**Netflix** (Consultant)**Next Gen Diagnostics** (Consultant)**PathoQuest** (Consultant)**PhAST** (Consultant)**Qvella** (Consultant)**Samsung** (Other Financial or Material Support, Patent Royalties)**Selux Diagnostics** (Consultant)**Shionogi & Co., Ltd.** (Grant/Research Support)**Specific Technologies** (Consultant)**TenNor Therapeutics Limited** (Grant/Research Support)**Torus Biosystems** (Consultant)**Up-to-Date** (Other Financial or Material Support, Honoraria) **Robin Patel, MD**, BioFire (Individual(s) Involved: Self): Grant/Research Support; Contrafect (Individual(s) Involved: Self): Grant/Research Support; IDSA (Individual(s) Involved: Self): Editor's stipend; NBME, Up-to-Date and the Infectious Diseases Board Review Course (Individual(s) Involved: Self): Honoraria; Netflix (Individual(s) Involved: Self): Consultant; TenNor Therapeutics Limited (Individual(s) Involved: Self): Grant/Research Support; to Curetis, Specific Technologies, Next Gen Diagnostics, PathoQuest, Selux Diagnostics, 1928 Diagnostics, PhAST, Torus Biosystems, Mammoth Biosciences and Qvella (Individual(s) Involved: Self): Consultant

